# Hyperferritinemia and the Risk of Liver Fibrosis and Liver-Related Events in Patients with Type 2 Diabetes Mellitus and Metabolic Dysfunction-Associated Steatotic Liver Disease

**DOI:** 10.3390/medicina61091518

**Published:** 2025-08-24

**Authors:** Simona Cernea, Andrada Larisa Roiban, Danusia Onișor

**Affiliations:** 1 Department M3/Internal Medicine I, George Emil Palade University of Medicine, Pharmacy, Science, and Technology of Târgu Mureş, 540142 Târgu Mureş, Romania; 2 Diabetes, Nutrition and Metabolic Diseases Outpatient Unit, Emergency County Clinical Hospital, 540136 Târgu Mureş, Romania; 3 Diabetes Compartment, Mediaș Municipal Hospital, 551030 Mediaș, Romania; andrada_pasc@yahoo.com; 4 Doctoral School of Medicine and Pharmacy, George Emil Palade University of Medicine, Pharmacy, Science, and Technology of Târgu Mureş, 540142 Târgu Mureş, Romania; 5 Department ME2/Internal Medicine VII, George Emil Palade University of Medicine, Pharmacy, Science and Technology of Târgu Mureş, 540142 Târgu Mureş, Romania; danusia.onisor@umfst.ro; 6 Gastroenterology Clinic, Mureș County Clinical Hospital, 540103 Târgu Mureş, Romania

**Keywords:** ferritin, Metabolic Dysfunction-Associated Steatotic Liver Disease (MASLD), T2DM, liver fibrosis, haptoglobin

## Abstract

*Background and Objectives:* This study evaluated the correlation between hyperferritinemia and markers of liver steatosis, fibrosis, and risk of liver-related events in patients with type 2 diabetes mellitus (T2DM) and Metabolic Dysfunction-Associated Steatotic Liver Disease (MASLD). *Material and Methods:* This study included 271 patients that underwent a comprehensive medical evaluation. Hyperferritinemia was defined by values >200 ng/mL (females) and >300 ng/mL (males). Liver fibrosis and steatosis were evaluated by several non-invasive indexes, and Liver Risk Score (LRS) was calculated to determine the risk of liver-related events. Their correlation with serum ferritin was investigated by bivariate and multiple regression analyses. Receiver Operating Characteristic (ROC) analyses were used to assess the accuracy to predict advanced fibrosis and increased LRS. Statistical significance was set at *p* < 0.05. *Results*: The median serum ferritin level was 94.4 [128.1] ng/mL. Metabolic hyperferritinemia was present in 12.54% of patients. Patients with hyperferritinemia had higher liver enzymes, HbA1c, HOMA-IR, and increased markers of liver steatosis and fibrosis, with a higher prevalence of advanced fibrosis (OR = 3.744 [1.481, 9.460], *p* = 0.0081). LRS was highest in patients with hyperferritinemia (7.99 ± 2.01 vs. 7.12 ± 1.32 vs. 6.54 ± 1.06, *p* < 0.0001). Serum ferritin levels were correlated with LRS (β = 0.190 [0.001; 0.003], *p* < 0.001), liver fibrosis (Fibrotic NASH Index) (β = 0.198 [0.000; 0.001], *p* < 0.001), and steatosis, while haptoglobin concentrations were correlated negatively with them. Serum ferritin predicted the moderate risk of liver-related outcomes with an acceptable performance (area under the ROC curve = 0.726 [0.590; 0.862], *p* = 0.001). *Conclusions*: Hyperferritinemia is associated with liver fibrosis and steatosis and a higher risk of liver-related events in patients with T2DM and MASLD.

## 1. Introduction

Type 2 diabetes mellitus (T2DM) and Metabolic Dysfunction-Associated Steatotic Liver Disease (MASLD) (formerly known as Non-Alcoholic Fatty Liver Disease (NAFLD)) are two interrelated chronic diseases that raise significant global health challenges. MASLD includes a range of liver conditions, from simple steatosis to steatohepatitis (MASH) without/with fibrosis (that can progress to cirrhosis), and hepatocellular carcinoma [1]. During progression towards advanced stages of liver disease, multiple local and systemic metabolic derangements occur, and identifying patients at higher risk of rapid progression remains a critical clinical imperative [2].

Ferritin is a protein found in higher concentrations in liver, spleen, and marrow macrophages that plays a key role in iron homeostasis by providing its intracellular storage in a bio-available form [3,4]. Apart from being a biomarker of iron metabolism, serum ferritin is also an indicator of chronic inflammation, oxidative stress (both contributing to liver injury), and metabolic dysfunction [4,5]. In fact, an intricate relationship between T2DM and iron metabolism has been described [6]. Dysregulated iron homeostasis can contribute to the pathogenesis of T2DM through reduced insulin secretion and insulin resistance [6].

Furthermore, serum ferritin has gained attention as a potential non-invasive predictor of liver injury and fibrosis progression [7]. A meta-analysis of 14 studies found elevated serum ferritin levels in subjects with MASLD, with higher values in the presence of steatohepatitis [8]. Recently, a prospective cohort study of 1895 participants reported that higher serum ferritin and its changing trajectory were associated with the risk of new-onset MASLD [9]. In addition, higher serum ferritin concentrations appear to be associated with the severity of liver disease in patients with MASLD [10,11]. A liver histology study found that serum ferritin levels greater than 1.5 times the upper limit of normal (ULN) were independently associated with more severe histologic features of MASLD, including more advanced hepatic fibrosis [10]. In a prospective multicenter study that monitored a cohort of 1342 biopsy-proven MASLD patients for 96 months, hyperferritinemia was associated with a higher risk of liver-related events and all-cause mortality [11].

Metabolic hyperferritinemia was recently defined by a Consensus Statement of an international panel of experts as increased serum ferritin levels (above sex-defined ULN) that reflect alterations in iron metabolism associated with metabolic dysfunction [12]. It also graded it into the following three stages: stage 1 (metabolic hyperferritinemia), stage 2 (dysmetabolic iron accumulation), and stage 3 (dysmetabolic iron overload syndrome), according to serum ferritin levels, iron stores, and organ damage related to iron [12].

In this study, we aimed to evaluate the correlation between hyperferritinemia and non-invasive markers of liver steatosis, fibrosis, and the risk of liver-related events in patients with T2DM and MASLD.

## 2. Materials and Methods

Study population: In this study, T2DM patients with MASLD were recruited from the Outpatient Unit of the Emergency County Clinical Hospital of Târgu Mureș and the Gastroenterology Department of the County Clinical Hospital, Târgu Mureș, Romania, for a period of 12 months (July 2022–July 2023).

Details regarding the materials and methods used in this larger study were previously published [13,14]. Briefly, the main inclusion criteria were a previous diagnosis of T2DM, age 30 years or more, and the presence of NAFLD [13]. At the time of study onset, the NAFLD definition was used (i.e., presence of liver steatosis/steatohepatitis in the absence of secondary causes of hepatic disease, including alcohol intake higher than ≥20 g/day for women and ≥30 g/day for men, respectively). However, in June 2023, there was a largely endorsed change in terminology (to MASLD) and its definition, and it was later shown that the two terms can be used interchangeably [15,16]. We therefore preferred to use the new terminology and definition to characterize the study population, since all our patients fulfilled the new definition criteria (presence of liver steatosis with at least one cardiometabolic risk factor, i.e., T2DM).

Clinical and laboratory evaluation: Several questionnaires were used to collect information regarding patients’ demographic and medical data. Vital signs and basic anthropometric parameters (weight, height, waist and hip circumferences) were obtained by standard methods. Additional anthropometric parameters were collected by using an InnerScan BC-545N segmental body composition monitor (Tanita, Tokyo, Japan) (i.e., % body fat, muscle mass, bone mass, segmental fat, and muscle mass). The presence of hepatic steatosis was confirmed by ultrasonographic (US) B-mode imaging (Hitachi Arietta v70 system, Mitsubishi Electric, Kyoto, Japan), which allowed estimation of the liver fat accumulation through the subjective assessment of liver brightness, the appearance of liver parenchyma, intrahepatic vessels, and diaphragm [17].

Fasting blood samples were obtained on the same day with the rest of the evaluations. The complete blood count (CBC) was analyzed shortly thereafter on a five-part differential automated hematology system (Mindray BC6200, India). Serum aliquots were stored at −80 °C for the subsequent analyses of the following parameters of interest: metabolic panel (glycated hemoglobin (HbA1c), blood glucose, C-peptide, lipid panel (total cholesterol, high-density lipoprotein (HDL) cholesterol, low-density lipoprotein (LDL) cholesterol, triglycerides), uric acid), liver panel (aspartate aminotransferase (ASAT), alanine aminotransferase (ALAT), gamma glutamyl transpeptidase (GGT), albumin, direct bilirubin), ferritin, haptoglobin, sex hormone-binding globulin (SHBG), and creatinine. Serum albumin, haptoglobin, and HbA1c were analyzed by using an immunoturbidimetric method, while the rest of the metabolic parameters, liver parameters, and creatinine were analyzed using a spectrophotometric method (Cobas Integra 400plus equipment, Roche Diagnostic, Mannheim, Germany). The analyses of serum ferritin, C-peptide, and SHBG were performed using a solid phase, two-site chemiluminescent immunometric assay (Immulite 2000 XPI system; Siemens Healthcare Diagnostics; Erlangen, Germany).

Metabolic hyperferritinemia was defined according to the recently published Consensus Statement, as follows: normal iron metabolism (serum ferritin levels: 50-ULN (upper limit of normal), i.e., 200 ng/mL for females, and 300 ng/mL for males), metabolic hyperferritinemia (stage 1: ULN-550 ng/mL, stage 2: 550–1000 ng/mL, and stage 3: >1000 ng/mL) [12].

Waist-to-hip ratio (WHR) and waist-to-height ratio (WHtR) were calculated, as well as body mass index (BMI) (weight/height^2^ (kg/m^2^)). Total body fat mass (BFM) (kg) was calculated from % body fat and weight. Appendicular muscle mass (AMM) was calculated as the sum of four-limbs muscle mass, and the indexed AMM (AMMi) was calculated as the ratio of AMM to BMI [18].

The insulin resistance was estimated by the Homeostatic Model Assessment (HOMA) for Insulin Resistance (HOMA-IR), calculated with the HOMA calculator version 2.2.3 [19]. The estimated glomerular filtration rate (eGFR) was computed using the CKD-EPI 2021 formula [20].

The following four indexes were calculated from the CBC data as surrogate markers of systemic inflammation: neutrophil-to-lymphocyte ratio (NLR), monocytes-to-lymphocytes ratio (MLR), systemic immune-inflammation index (SIII) (platelet count × neutrophil count/lymphocyte count), and systemic inflammatory response index (SIRI) (neutrophil count × monocyte count/lymphocyte count) [21,22].

In addition to the US evaluation, the following two non-invasive biomarkers of liver steatosis were calculated: Fatty Liver Index (FLI) = (e^0.953 × loge(TG) + 0.139 × BMI + 0.718 × loge(GGT) + 0.053 × waist − 15.745^)/(1 + e^0.953 × loge(TG) + 0.139 × BMI + 0.718 × loge(GGT) + 0.053 × waist − 15.745^) × 100 (30 rules out and ≥60 rules in steatosis), and the Index of NASH (non-alcoholic steatohepatitis) (ION) = 1.33 × WHR + 0.03 × triglycerides (mg/dL) + 0.18 × ALAT (U/L) + 8.53 × HOMA-IR − 13.93 (for men), and ION = 0.02 × triglycerides (mg/dL) + 0.24 × ALAT (U/L) + 9.61 × HOMA-IR − 13.99 (for women) (<11 excludes steatosis, >22 indicates steatosis, and >50 predicts NASH) [23,24].

Liver fibrosis was estimated using the following three validated non-invasive biomarkers: Fibrosis-4 (FIB-4) index = age (years) × ASAT (U/L)/[platelets (10^9^/L) × ALAT^1/2^ (U/L)] (<1.3 rules out advanced fibrosis, >2.67 rules in advanced fibrosis (F ≥ 2), while FIB-4 values between 1.3 and 2.67 are indeterminate), Fibrotic NASH Index (FNI) = e^(−10.33 + 2.54 × lnASAT (U/L) + 3.86 × lnHbA1c (%) − 1.66 × lnHDL (mg/dL)^/1 + e^(−10.33 + 2.54 × lnASAT (U/L) + 3.86 × lnHbA1c (%) − 1.66 × lnHDL (mg/dL)^ (with a rule-in and -off cutoff of 0.10), and APRI (ASAT to Platelet Ratio Index) [25,26,27]. To allow a better selection of patients with a higher risk of advanced fibrosis and without fibrosis, respectively, study patients were divided into the following three groups, based on a combination of two fibrosis indexes: both FIB-4 > 2.67 and FNI > 0.1 (advanced fibrosis), both FIB-4 < 1.3 and FNI ≤ 0.1 (no significant fibrosis), and the rest of the subjects were classified as having an indeterminate risk of advanced fibrosis.

The Liver Risk Score (LRS) was calculated online from the following parameters: age, sex, fasting blood glucose, total cholesterol, ASAT, ALAT, GGT, and platelets (available online at https://www.liverriskscore.com, accessed on December 2024). LRS was developed and validated as a prognostic score for long-term liver-related outcomes (liver cancer, liver-related hospitalization, and mortality) and significant liver fibrosis [28]. The score categorizes patients in the following categories: high risk (≥15), moderate risk (10–<15), low risk (6–<10), and minimal risk (<6).

Descriptive and inferential statistics were used for data analysis. The normality of data was verified with the Kolmogorov–Smirnov test. Normally distributed parameters are presented as mean ± standard deviation (SD), and the non-parametric data are presented as the median and interquartile range [IQR]. The comparisons between continuous variables were performed using the One-way ANOVA with the Tukey post-test (for normally distributed data) or the Kruskal–Wallis test with the Dunn post-test (for variables with non-Gaussian distribution). Fisher’s exact test was employed for the analysis of categorical variables, and odds ratios (ORs) [95% confidence interval (CI)] are reported. Spearman’s test was used to verify the association between two sets of variables without Gaussian distribution, Pearson’s test was used for normally distributed data, and results are presented as r [95% CI]. The multiple regression analyses were employed to test independent associations between variables of interest (serum ferritin, Liver Risk Score, FNI, and ION) and other relevant variables.

Additional sensitivity analyses were performed to identify the optimal threshold values for ferritin and the ferritin-to-haptoglobin ratio (FHR) that predict long-term liver outcomes (LRS) and advanced liver fibrosis by using receiver operating characteristics (ROC) analyses and Youden’s index. To identify significant liver fibrosis, values of 0.10 (for FNI) and of 2.67 (for FIB-4), respectively, were used. An LRS threshold ≥ 10 was used to identify moderate/high risk, and ≥6 was used for low/moderate/high risk. The areas under the ROC curve (AUROC) were calculated to assess the discrimination performance (value 1 indicating perfect performance). Statistical analysis was performed using GraphPad InStat3 software and IMB SPSS stat version 31.0.0.0. Statistical significance was assumed at *p* < 0.05.

## 3. Results

### 3.1. Baseline Characteristics

This study enrolled 278 patients with T2DM with MASLD, of whom seven met the exclusion criteria (i.e., other causes of chronic liver disease (including higher alcohol intake), malignant diseases in the last 5 years, autoimmune diseases). Thus, the analysis included data from 271 T2DM patients (mean age: 65.0 ± 8.4 years; mean T2DM duration: 9.7 ± 5.0 years; median BMI: 33.7 [7.68] kg/m^2^; median HbA1c: 6.8 [0.9]%). The relevant concomitant therapy is presented in Supplementary Table S1.

The median serum ferritin levels were 94.4 [128.1] ng/mL in this study group (63.4 [88.45] ng/mL for females and 149.5 [151.83] ng/mL for males). Among all subjects, 12.54% had metabolic hyperferritinemia (stage 1 (n = 29) and stage 2 (n = 5), which were grouped together in the present analysis), and none had serum ferritin values >1000 ng/mL. The clinical and laboratory characteristics were analyzed according to the following three categories: hyperferritinemia (>ULN), normal ferritin levels (50 ng/mL—ULN), and low ferritin levels (<50 ng/mL), and they are presented in Table 1. More male patients had hyperferritinemia, while more females had lower ferritin levels (Table 1, *p* < 0.0001 for trend). Patients with metabolic hyperferritinemia had higher ASAT, ALAT, GGT, and HbA1c compared to subjects with normal ferritin levels, as well as increased markers of liver steatosis and fibrosis (Table 1). In a subsequent analysis, the C-peptide levels and HOMA-IR were higher in patients with hyperferritinemia compared to the rest of the subjects (3.98 ± 1.57 ng/mL vs. 3.33 ± 1.63 ng/mL (3.04 [2.01] ng/mL), *p* = 0.0121 for C-peptide and 3.45 ± 1.51 vs. 2.82 ± 1.41 (2.65 [1.72]), *p* = 0.0113 for HOMA-IR, respectively). Subjects with lower serum ferritin concentrations (<50 ng/mL) had longer diabetes duration, lower AMMi, direct bilirubin, serum creatinine, triglycerides, SHBG, red blood cell parameters, and platelet count compared to subjects with normal ferritin levels (Table 1). Instead, there were no differences between the groups with regards to inflammatory indexes, suggesting the presence of iron overload rather than inflammation in patients with hyperferritinemia.

In the metabolic hyperferritinemia group, a higher percentage of subjects had markers of advanced fibrosis (assessed by the combination of FNI and FIB-4, as described in the Materials and Methods section), as follows: 21.05% versus 6.7% and 9.59% in the other two groups, respectively (*p* = 0.0104). This group had a significantly higher risk of advanced fibrosis compared to patients with normal/low ferritin levels (OR = 3.744 [95% CI: 1.481, 9.460], *p* = 0.0081).

**Figure 1:**
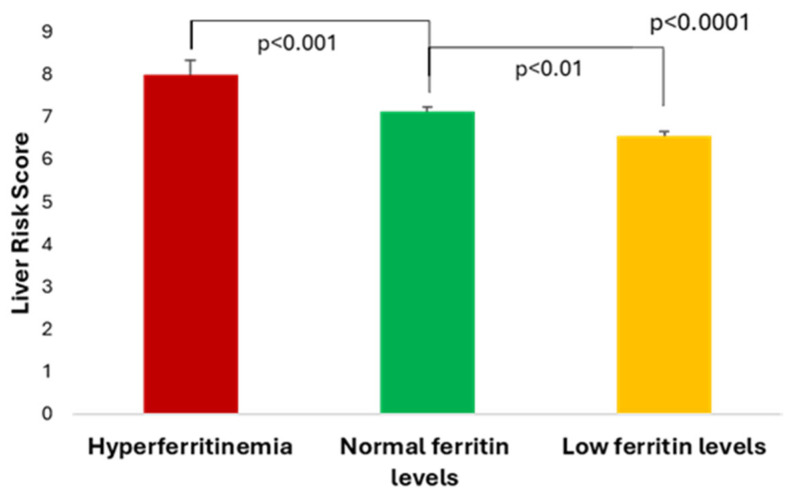
The Liver Risk Score according to the three serum ferritin categories.

Out of all of them, 54 patients (19.9%) had minimal risk to develop future liver-related events (as predicted by LRS), 205 (75.6%) had low risk, while 12 patients (7.7%) had moderate risk (none of the patients had high risk, i.e., LRS ≥15). The mean LRS was higher in subjects with metabolic hyperferritinemia versus patients with normal ferritin levels, while patients with ferritin levels <50 ng/mL had lower LRS values (7.99 ± 2.01 (7.58 [2.48]) vs. 7.12 ± 1.32 (6.89 [1.31]) vs. 6.54 ± 1.06; *p* < 0.0001) (Figure 1).

The correlations between serum ferritin levels and liver parameters, as well as other clinical and biological parameters, were investigated by bivariate analyses. Significant correlations were noted for the parameters presented in Table 2, while for the rest of them, no significant associations were observed, although for fasting blood glucose, the non-significance was borderline (r = 0.12 [−0.01; 0.24], *p* = 0.0543).

In the multivariable analysis (dependent variables: age, diabetes duration, diastolic blood pressure, WHR, AMMi, serum albumin, ALAT, ASAT, GGT, direct bilirubin, SHBG, HOMA-IR, creatinine, hemoglobin, and platelets), serum ferritin levels were correlated independently with diabetes duration, liver enzymes (ASAT, GGT), AMMi, and hemoglobin (Table 3a), while for the rest of the variables, the correlations were non-significant.

To further understand the impact of ferritin on liver health/liver-related outcomes, several secondary multivariable analyses were performed. LRS, FNI, and ION, respectively, were the dependent variables, while several parameters known to be associated with the risk of liver disease progression were used as independent variables, along with serum ferritin. For the LRS analysis, the independent variables were BMI, systolic blood pressure, diabetes duration, HbA1c, triglycerides, ferritin, haptoglobin, direct bilirubin, creatinine, and AMMi (parameters used to calculate the score were not included). Serum ferritin levels, HbA1c, direct bilirubin, BMI, and AMMi were positively correlated with LRS, while haptoglobin was negatively correlated with it (Table 3b), and for the rest of the parameters, no significant correlations were observed. For the fibrosis risk (FNI) analysis, the independent variables were age, sex, BMI, systolic blood pressure, HOMA-IR, triglycerides, GGT, ferritin, haptoglobin, and eGFR (parameters used to calculate FNI were not used). FNI was positively correlated with serum ferritin, HOMA-IR, and GGT, and haptoglobin was negatively associated with it (Table 3c), while for the other independent variables, the correlations were not significant. For the liver steatosis (ION) analysis, the independent variables were the same, except that HOMA-IR and triglycerides (used to calculate ION) were replaced with HbA1c. In this analysis, hepatic steatosis (as evaluated by ION) was positively correlated with serum ferritin and BMI, and negatively correlated with age and eGFR (Table 3d), while the remaining variables were not significantly associated with it.

### 3.2. Serum Ferritin and Haptoglobin Levels in Relationship with Liver Risk Score and Liver Fibrosis Markers

**Figure 2:**
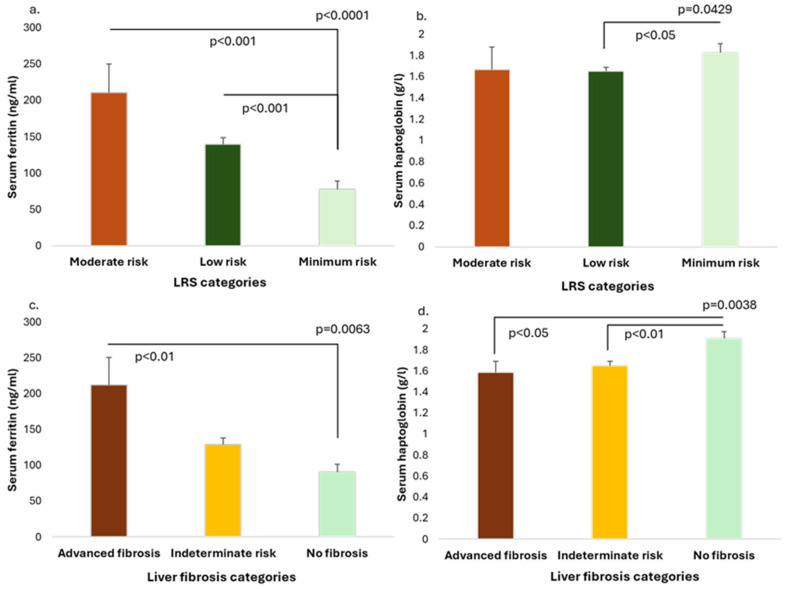
(**a**) Serum ferritin levels and (**b**) serum haptoglobin according to the Liver Risk Score categories; and (**c**) serum ferritin and (**d**) serum haptoglobin concentrations according to the liver fibrosis risk categories.

The serum ferritin levels were compared between the three LRS categories, and progressively increasing ferritin values with higher LRS category (minimal vs. low vs. moderate risk) were observed, as follows: 77.57 ± 85.96 (42.30 [87.95]) ng/mL vs. 139.59 ± 132.72 (106.00 [124.0]) ng/mL vs. 210.78 ± 137.71 ng/mL, *p* < 0.0001 (Figure 2a). In addition, patients in the minimal risk category had higher haptoglobin levels (1.83 ± 0.55 g/L vs. 1.65 ± 0.60 (1.61 [0.79]) g/L vs. 1.67 ± 0.73 (1.50 [0.87]) g/L, *p* = 0.0429) (Figure 2b).

There was a progressive increase of the serum ferritin levels in the three liver fibrosis categories (defined by the combination of FIB-4 and FNI indexes), as follows: 90.34 ± 79.32 (63.40 [82.90]) ng/mL (no fibrosis group) vs. 129.48 ± 122.83 (95.60 [115.43]) ng/mL (indeterminate risk) vs. 212.17 ± 194.12 ng/mL (advanced fibrosis group), *p* = 0.0063, with the advanced fibrosis group having significantly higher ferritin values compared to the group without fibrosis (*p* < 0.01) (Figure 2c). Furthermore, patients with advanced fibrosis and with indeterminate risk presented lower haptoglobin levels compared to patients without liver fibrosis, as follows: 1.59 ± 0.51 (1.54 [0.54]) g/L vs. 1.65 ± 0.63 g/L vs. 1.91 ± 0.44 g/L, *p* = 0.0038 (Figure 2d).

As hyperferritinemia appeared to be associated with a higher risk of liver fibrosis and liver-related outcomes, while the groups without fibrosis and minimum risk of liver outcomes had higher haptoglobin levels, the study population was further divided into the following four categories to better explore the relationship between liver health and ferritin and haptoglobin levels: hyperferritinemia and normal haptoglobin (group 1, n = 24), hyperferritinemia and high haptoglobin levels (>2.0 g/L) (group 2, n = 10), normal/low ferritin and normal haptoglobin levels (group 3, n = 171), and normal/low ferritin levels and high haptoglobin levels (group 4, n = 66). Hyperferritinemia with normal haptoglobin levels was associated with higher LRS and FNI, while normal/low ferritin levels and high haptoglobin levels were associated with lower FNI and LRS (Supplementary Figure S1).

**Figure 3:**
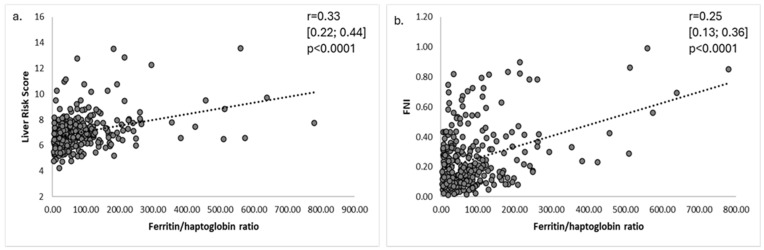
Correlation between the ferritin-to-haptoglobin ratio and (**a**) Liver Risk Score, and (**b**) FNI (FNI= Fibrotic NASH Index; data are correlation coefficient r [95% confidence interval (CI)]).

Furthermore, the FHR (one outlier value of 8570.0 was excluded) correlated positively with both LRS (r = 0.33 [95% CI: 0.22; 0.44], *p* < 0.0001) and FNI (r = 0.25 [95% CI: 0.13; 0.36], *p* < 0.0001) (Figure 3a,b).

### 3.3. Performance of Ferritin and FHR to Predict Advanced Liver Fibrosis and Liver-Related Outcomes

**Figure 4:**
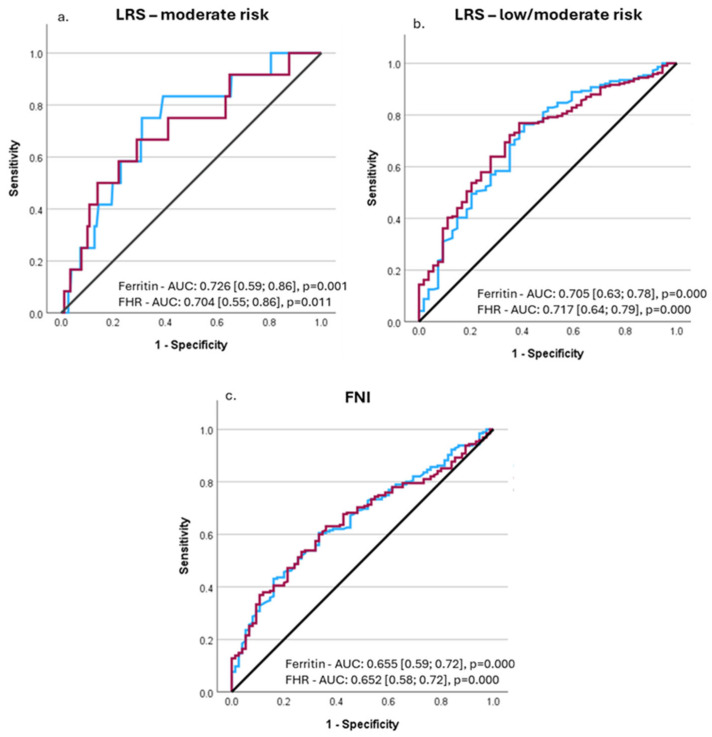
Area under the curve (AUC) receiver operating characteristics (ROC) for serum fibrosis and FHR (ferritin to haptoglobin ratio) for the diagnosis of (**a**) the moderate risk of liver-related outcomes (assessed by LRS), (**b**) low/moderate risk of liver-related outcomes (assessed by LRS), and (**c**) significant fibrosis (assessed by FNI) (LRS = Liver Risk Score, FNI= Fibrotic NASH Index; FHR = ferritin-to-haptoglobin ratio; blue lines are for ferritin, red lines for FHR).

The performance of serum ferritin concentrations and FHR (without the outlier) to predict liver-related outcomes and advanced liver fibrosis was investigated by means of ROC analysis. Serum ferritin concentrations predicted the moderate risk of liver-related outcomes (LRS > 10) with an AUROC of 0.726 [95% CI: 0.590; 0.862], *p* = 0.001, while for the FHR, the AUROC was 0.704 [95% CI: 0.547; 0.861], *p* = 0.011 (Figure 4a). Serum ferritin values of 117 ng/mL predicted a moderate risk of liver-related outcomes with a sensitivity of 83.3% and specificity of 60.9% (Youden’s index = 0.442), while the FHR of 94.56 had a sensitivity of 66.7% and a specificity of 70.9% (Youden’s index = 0.376). The sex-based analyses resulted in the same optimal cutoff values in females, both for ferritin and FHR, but the AUROCs were lower (0.706 and 0.626, respectively), and statistical significance was not reached. In men, however, the optimal cutoffs were higher (146.5 ng/mL for ferritin and 115.35 for FHR) but with lower AUROCs (0.690 and 0.681, respectively) and without statistical significance.

In a subsequent analysis, we investigated the performance of ferritin and FHR to predict the low and moderate risk of liver-related outcomes (LRS > 5). For ferritin, the AUROC was 0.705 [95% CI: 0.625; 0.784], *p* = 0.000, while for FHR, it was 0.717 [95% CI: 0.644; 0.791], *p* = 0.000 (Figure 4b). The optimal ferritin cutoff to predict the low/moderate risk of liver-related events was 54.35 ng/mL (sensitivity: 76.4%, specificity: 59.3%), while the FHR value of 31.70 had a specificity of 76.9% and sensitivity of 61.1%. The sex-based analyses indicated the same optimal cutoffs both for ferritin and FHR (AUROC = 0.643 [95% CI: 0.545; 0.741], *p* = 0.004, and 0.655 [95% CI: 0.563, 0.747], *p* = 0.001) for women. Instead, for men, the best predictive cutoff was 92.8 ng/mL for ferritin and 32.68 for FHR, but the AUROCs were lower (0.638 and 0.658, respectively) and did not reach statistical significance.

Finally, we used the same approach to investigate the performance to predict advanced fibrosis (as assessed by FNI and FIB-4). Ferritin had an AUROC of 0.655 [95% CI: 0.587; 0.724], *p* = 0.000, while for FHR, it was 0.652 [95% CI: 0.584; 0.720], *p* = 0.000 (Figure 4c.). The highest Youden’s index (0.272) was noted for a ferritin value of 84.90 ng/mL (sensitivity: 60.5%, specificity 72.8%), and for a FHR of 52.55 (Youden’s index = 0.271, sensitivity: 63.1%, and specificity: 64.0%). Lower performance was obtained in relation to FIB-4 for both variables (*p* = NS). The AUROC was 0.605 [95% CI: 0.482; 0.729], *p* = 0.095 (for ferritin), and 0.605 [95% CI: 0.477; 0.733], *p* = 0.109 (for FHR).

## 4. Discussion

The present study found that T2DM patients with MASLD and metabolic hyperferritinemia had higher markers of liver steatosis and fibrosis (with an almost four-fold higher risk of advanced fibrosis), worse long-term glycemic control, and higher insulin resistance. They also had higher values of LRS, which was previously shown to predict liver fibrosis (better than other indexes), long-term liver related outcomes, and diabetes-related mortality [28,29]. Serum ferritin was positively correlated with liver enzymes, LRS, FNI, and ION (markers of liver fibrosis and steatosis), while haptoglobin had a negative association with LRS and FNI in the multivariable analyses. Subjects with higher ferritin and normal haptoglobin levels had a higher risk of fibrosis compared to those with normal/low serum ferritin levels and high haptoglobin levels. Furthermore, the FHR presented a weak positive correlation with both FNI and LRS. Finally, we found that a ferritin cutoff value of 54.35 mg/dL predicted the low/moderate risk of future liver-related events, while the cutoff value of 117 ng/mL predicted the moderate risk of liver-related events (both with moderate/good sensitivity, but lower specificity), although higher values appeared more relevant for males.

Ferritin is an iron-storage protein that plays a role in the acute-phase response to inflammation and injury [30,31]. It is produced by hepatocytes and activated macrophages (including Kupffer cells) in response to inflammatory signals, and it is also released by damaged liver cells [31,32,33]. The increased release of ferritin further exacerbates inflammation, as it has been shown in animal models that it may activate the mitogen-activated protein kinase (MAPK)-triggered NF-κB in the hepatic stellate cells (HSCs) in an iron-independent manner, subsequently increasing the expression of pro-inflammatory mediators [31,34]. The inflammatory activity of the hepatic macrophages further activates the HSCs, which then produce extracellular matrix contributing to liver fibrosis [2,35]. In addition, it appears that the activated HSCs express an H-ferritin receptor through which the ferritin released by the Kupffer cells (following the processing of senescent red blood cells) is internalized [36,37,38]. The accumulation of iron further favors the development of fibrosis through the activation of oxidative stress via Fenton reaction [39]. Thus, hyperferritinemia may contribute to liver fibrosis, and in fact, similar to our data, other studies showed that serum ferritin was correlated with liver fibrosis (and the presence of MASLD), mainly in the context of concomitant diabetes mellitus [9,40,41,42]. In a different approach, Amangurbanova M. and collaborators recently confirmed the correlation between hyperferritinemia and significant fibrosis in subjects with T2DM [42]. They have shown that about 80% of T2DM patients with hyperferritinemia have MASLD and that hyperferritinemia was an independent predictor of significant fibrosis (assessed by magnetic resonance elastography) (OR = 2.33, *p* = 0.001) [43].

We have shown that T2DM patients with MASLD and metabolic hyperferritinemia, in addition to having higher levels of liver enzymes, worse metabolic control, and increased markers of liver steatosis and fibrosis, also presented higher LRS values, indicative of a higher risk of future liver-related events, further substantiated by the positive correlation between serum ferritin levels and LRS. Although LRS is a non-invasive biomarker that predicts the risk of liver-related outcomes (i.e., significant fibrosis, liver-related hospitalization, liver cancer, and liver-related mortality), and even if we could not employ an event-driven analysis, our data are in accordance with a couple of recent studies demonstrating a significant association between metabolic hyperferritinemia and liver-related events in patients with steatotic liver disease [11,44,45]. The study by Armandi A. et al. showed that hyperferritinemia was associated with a 50% higher risk of liver-related events and a 27% higher risk of all-cause mortality in 1342 MASLD patients followed up for 96 months [11]. Similarly, Suresh D. and colleagues showed, in a retrospective study of 7333 patients with MASLD, that hyperferritinemia was associated with an increased incidence of liver-related events (HR = 1.92, *p* = 0.019) and mortality (HR = 1.68, *p* < 0.001) [44]. Finally, in a large cohort of subjects with steatotic liver disease (of whom 15,744 had MASLD) followed for a median of 12.3 years, patients with hyperferritinemia had an increased risk of liver-related events (i.e., development of decompensation (ascites, variceal bleeding, hepatic encephalopathy) or hepatocellular carcinoma) (adjusted HR = 2.69) [45].

Furthermore, several studies have evaluated the accuracy of ferritin to predict hepatic fibrosis, with mixed results. By analyzing the National Health and Nutritional Examination Survey 2017–2020 (NHANES 2017–2020) data, Ramasamy J. concluded that serum ferritin was a poor predictor of liver fibrosis (defined by liver stiffness measurement (LSM) >8 kPa) (AUC = 0.59, sensitivity = 53.5%, specificity = 60.3%) in subjects with NAFLD [46]. Trasolini R. and colleagues also reported, in a cohort of 224 patients with NAFLD (and a mean LSM of 5.2 kPa), that serum ferritin was not predictive of significant liver fibrosis (AUROC = 0.54; CI and *p* value not reported) [47]. On the contrary, Hanafi AS et al. found that ferritin was a good predictor of biopsy-proven significant fibrosis (AUROC = 0.809, *p* = 0.001), with a sensitivity of 95.8% and a specificity of 90% for the cutoff value of 321 ng/dL [48]. Although not reporting AUROC, specificity, and sensitivity data, Kowdley KV et al. showed, in a liver biopsy study (n = 628 adults with NAFLD), that serum ferritin levels > 1.5 × ULN (i.e., >300 ng/mL in women and >450 ng/mL in men) were independently associated with advanced fibrosis (OR = 1.66, *p* = 0.028) [10]. Our data also indicated a poor discrimination ability of serum ferritin to predict significant fibrosis (as assessed by non-invasive indexes, FNI, and FIB-4). However, we found a fair discrimination ability of serum ferritin to predict a moderate risk of liver-related outcomes, with a value of 117 ng/mL having a good sensitivity (83.3%, indicating a good ability to identify at-risk individuals) but with a lower specificity, although the results suggest that sex-based thresholds should be defined. A lower ferritin cutoff of 54.35 ng/mL predicted low and moderate risk of liver-related events, a value close to that reported by El Nakeeb N. et al. in a small histology study (i.e., 51.95 ng/mL) that predicted liver fibrosis [7].

Although serum ferritin alone might not be an ideal marker to predict liver-related outcomes, combining it with other parameters (i.e., liver enzymes, HOMA-IR) or non-invasive scoring systems (i.e., FIB-4, NFS) improves its prediction value [9,47]. In a recent paper, Qadri S. and Yki-Järvinen H. argued, based on simple biostatistical inference, that the currently recommended screening pathways to detect advanced liver fibrosis in subjects with T2DM result in high-rate false-positive referrals to hepatology [49]. These screening algorithms are based on FIB-4 calculation, and transient elastography/Enhanced Liver Fibrosis test are recommended as subsequent evaluations for individuals at risk [49]. However, the latter tests are not largely available in all countries; therefore, we propose here that other non-invasive markers such as ferritin (that is widely available and not expensive) might be used as an intermediate-step evaluation (perhaps in a combined score/algorithm).

We have explored the ability of the FHR to predict liver fibrosis and liver-related events (LRS), and the results demonstrated an overall comparable performance with ferritin alone (slightly better for low/moderate LRS). While the FHR has not been used before, other composite scores that include ferritin or haptoglobin individually (e.g., FibroTest, FibroMeterNAFLD) show improved diagnostic accuracy [50]. Haptoglobin is an antioxidant scavenger glycoprotein synthesized by the liver that binds free hemoglobin to prevent oxidative damage and is also involved in other processes like inflammation and immune regulation [51]. In chronic liver disease, haptoglobin levels may decrease. We have shown here that haptoglobin levels were lower in patients with advanced liver fibrosis compared to patients without markers of fibrosis, and they correlated independently in a negative manner with FNI and LRS, which is in accordance with previously published data. In a small liver biopsy study (n = 92 patients with viral and autoimmune hepatitis), haptoglobin was negatively correlated with liver fibrosis stage (r = −0.28, *p* < 0.01) [52]. Another Korean study that included 150 patients with chronic liver disease (mostly viral hepatitis and 9.5% liver steatosis) who underwent liver biopsy reported a progressive decrease in haptoglobin levels with increasing fibrosis stage and a significant negative correlation between haptoglobin and liver fibrosis stage (r = −0.50, *p* < 0.001) [53]. Furthermore, we found that patients with low/normal ferritin levels but high haptoglobin had lower FNI and LRS. One possible explanation for this finding is provided by Zhang A. et al., who performed a proteomic network analysis study in mice with fibrosis and showed that haptoglobin expression was upregulated in the early stages following toxic fibrosis induction, highlighting a possible compensatory role in early liver injury [54]. The authors suggested that haptoglobin increase may precede the onset and progression of fibrosis, and perhaps, the concentrations further decline during chronic disease progression [54]. Nevertheless, this hypothesis should be tested in further prospective studies.

There are several limitations of the study. The gold-standard method for the assessment of liver steatosis and fibrosis (liver biopsy), which would have made possible the evaluation of iron liver depots as well, could not be employed. However, we used well-validated and largely accepted non-invasive indexes. In addition, we combined two fibrosis indexes to better select patients with advanced fibrosis. Secondly, we did not have longitudinal ferritin (and haptoglobin) data, which limits causal inferences between liver health status and changes in iron metabolism (ferritin and haptoglobin levels). Future studies should explore the long-term changes of iron metabolism (in blood and liver) as MASLD progresses to more advanced stages, as well as the pathogenetic mechanisms involved. Perhaps a larger sample size would have allowed subgroup analysis and a better identification of sex-based cutoffs for ferritin to detect liver-related outcomes, providing more generalizable results. Furthermore, the observational design of the study precluded us from addressing all confounding factors that might influence the outcomes.

Nevertheless, this study brings the argument for integrating serum ferritin into the non-invasive stepwise evaluation of MASLD patients for advanced fibrosis (with risk stratification), especially those with T2DM. This is particularly relevant in clinical settings where more expensive non-invasive imaging methods are not widely available for first-hand screening and liver biopsies are not routinely performed. The findings advocate for the screening of serum ferritin (and perhaps haptoglobin) in T2DM patients with MASLD to identify those at higher risk of liver disease progression. Future histology studies are needed to validate the suggested approach, allowing a better selection of high-risk patients that need to undergo liver biopsy.

## 5. Conclusions

Hyperferritinemia is associated with liver fibrosis and steatosis and a higher risk of liver-related events in patients with T2DM and MASLD. Serum haptoglobin is negatively correlated with markers of liver fibrosis. Serum ferritin predicts, with fair performance, the risk of liver-related outcomes, but sex-based thresholds should be defined.

## Figures and Tables

**Table 1:** Clinical and laboratory parameters, and inflammatory and liver indexes in study patients according to the three serum ferritin level categories (data are presented as mean ± SD for normally distributed data and median [IQR] for non-parametric data; F = female; M = male; BP = blood pressure; BMI = body mass index; AMMi = indexed appendicular muscle mass; WHtR = waist-to-height ratio; BG = blood glucose; HbA1c = glycated hemoglobin; HDL = high-density lipoprotein; LDL = low-density lipoprotein; HOMA-IR = Homeostatic Model Assessment for Insulin Resistance; ASAT = aspartate aminotransferase; ALAT = alanine aminotransferase; GGT = gamma glutamyl transpeptidase; SHBG = sex hormone-binding globulin; eGFR = estimated glomerular filtration rate; MCV = mean corpuscular volume; MCH = mean corpuscular hemoglobin; MCHC = mean corpuscular hemoglobin concentration; NLR = neutrophil-to-lymphocyte ratio; MLR = monocytes-to-lymphocytes ratio; SIII = systemic immune-inflammation index; SIRI = systemic inflammatory response index; FLI = Fatty Liver Index; ION = Index of NASH (Non-alcoholic steatohepatitis); FIB-4 = Fibrosis-4 index; FNI = Fibrotic NASH Index; APRI = ASAT-to- Platelet Ratio Index; ^ one outlier was excluded; * *p* < 0.05; ** *p* < 0.01; *** *p* < 0.001).

	Hyperferritinemia (>ULN) n = 34	Normal Ferritin Levels (50-ULN) n = 163	Low Ferritin Levels (<50 ng/mL) n = 74	*p*
Serum ferritin (ng/mL)	335.0 [189.0]	115.0 [80.3]	26.84 ± 11.67	<0.0001
Clinical and anthropometric parameters				
Age (years)	63.56 ± 8.93	66.0 [10.0]	65.77 ± 8.40	0.4034
Diabetes duration (years)	8.03 ± 4.59 **	9.0 [6.0] *	11.0 [5.0] *,**	0.0029
Sex (F/M) (number (%))	12 (35.3)/22 (64.7)	79 (48.5)/84 (51.5)	58 (78.4)/16 (21.6)	<0.0001
Systolic BP (mmHg)	134.94 ± 14.03	135.46 ± 15.88	133.75 [17.5]	0.6402
Diastolic BP (mmHg)	84.10 ± 9.10	82.00 [12.50]	80.0 [12.13]	0.0725
BMI (kg/m^2^)	33.48 ± 5.17	33.76 [7.06]	34.39 ± 6.13	0.7839
AMMi	0.79 [0.31] **	0.75 [0.29] **	0.67 [0.17] **,**	0.0002
Fat mass (kg)	33.45 ± 10.67	32.09 [12.98]	31.41 [14.22]	0.8769
WHtR	0.68 ± 0.07	0.68 ± 0.07	0.69 ± 0.09	0.3274
Waist circumference (cm)	113.74 ± 10.24	111.91 ± 11.43	110.22 ± 12.72	0.3214
Hip circumference (cm)	109.21 ± 10.37	107.0 [14.00]	109.51 ± 11.28	0.9082
Laboratory parameters				
Fasting BG (mg/dL)	137.95 [37.05]	138.63 [30.59]	135.35 [32.27]	0.1591
HbA1c (%)	7.05 [0.80] *	6.80 [0.9] *	6.8 [0.85]	0.0433
Total cholesterol (mg/dL)	153.02 [49.19]	157.16 [45.97]	150.42 (45.65]	0.2878
HDL cholesterol (mg/dL)	44.57 ± 10.11	43.85 [11.45]	47.22 ± 10.96	0.1593
LDL cholesterol (mg/dL)	88.41 ± 34.43	85.73 [39.37]	77.32 [40.36]	0.1750
Triglycerides (mg/dL)	132.38 [96.31]	163.98 [98.34] *	132.95 [77.36] *	0.0289
Uric acid (mg/dL)	5.78 ± 1.49	5.89 [1.86]	5.60 ± 1.37	0.1332
C-peptide (ng/mL)	3.98 ± 1.57 **	3.32 [2.07] **	2.79 ± 1.28 **,**	<0.0001
HOMA-IR	3.45 ± 1.51 ***	2.82 [1.82] ***	2.35 ± 1.09 ***,***	<0.0001
Albumin (g/dL)	4.68 ± 0.25	4.66 ± 0.24	4.61 ± 0.21	0.4773
ALAT (U/L)	24.20 [27.18] ***	18.81 [16.19] ***,***	13.69 [10.98] ***	<0.0001
ASAT (U/L)	27.54 [23.05] *,**	20.79 [9.95] *	19.08 [7.33] **	0.0024
GGT (U/L)	49.09 [44.28] *,***	29.90 [25.02] *,***	21.98 [20.92] ***,***	<0.0001
Direct bilirubin (mg/dL)	0.23 ± 0.08 **	0.21 [0.10] ***	0.16 [0.09] **,***	0.0001
Haptoglobin (g/L)	1.63 ± 0.59	1.64 ± 0.57	1.81 ± 0.65	0.1152
SHBG (nmol/L)	33.67 ± 14.44	32.00 [18.70] *	37.45 [20.88] *	0.0153
Creatinine (mg/dL)	0.88 ± 0.18	0.85 [0.27] *	0.75 [0.25] *	0.0078
eGFR (ml/min/1.73m^2^)	93.86 [66.20]	89.96 [21.30]	91.26 [27.58]	0.5293
Red blood cell count (10^6^/μL)	4.99 ± 0.44 *	4.81 ± 0.52	4.70 ± 0.51 *	0.0209
Hemoglobin (g/dL)	15.01 ± 1.41 ***	14.44 ± 1.47 ***	13.30 [1.80] ***,***	<0.0001
Hematocrit (%)	45.24 ± 3.98 ***	43.62 ± 4.28 ***	41.35 ± 4.53 ***,***	<0.0001
MCV (fL)	91.5 [7.98] **	90.9 [5.10] ***	88.65 [4.80] **,***	0.0001
MCH (pg)	30.15 ± 2.55 ***	30.11 ± 1.72 ***	28.9 [2.13] ***,***	<0.0001
MCHC (g/dL)	33.18 ± 0.65 **	33.1 [0.90] ***	32.54 ± 0.92 **,***	<0.0001
Leucocyte count (10^3^/μL)	7.88 ± 1.76	7.53 ± 1.83	7.86 ± 2.0	0.3434
Neutrophil count (10^3^/μL)	4.59 ± 1.39	4.28 [1.71]	4.78 ± 1.60	0.4493
Lymphocyte count (10^3^/μL)	2.51 ± 0.66	2.24 ± 0.69	2.32 ± 0.67	0.1130
Monocyte count (10^3^/μL)	0.50 ± 0.15	0.45 [0.17]	0.48 ± 0.13	0.5361
Eosinophil count (10^3^/μL)	0.21 [0.15]	0.18 [0.16]	0.18 [0.18]	0.6626
Basophil count (10^3^/μL)	0.04 [0.03]	0.04 [0.02]	0.04 [0.02]	0.5284
Platelet count (10^3^/μL) ^	226.97 ± 48.86 *	228.28 ± 59.40 **	258.02 ± 75.95 *,**	0.0028
Inflammatory indexes				
NLR	1.93 ± 0.67	1.96 [1.04]	1.92 [1.17]	0.4001
SIII	436.28 ± 174.28	431.63 [277.30]	519.46 [326.18]	0.0720
SIRI	0.97 ± 0.46	0.89 [0.58]	0.91 [0.73]	0.7737
MLR	0.21 ± 0.07	0.20 [0.10]	0.20 [0.11]	0.5533
Liver steatosis and fibrosis indexes				
FLI	91.60 [21.25]	91.00 [18.6]	81.60 [31.00]	0.0399
ION	28.55 ± 14.68 ***	21.17 [18.75] ***,***	15.42 ± 10.97 ***	<0.0001
FIB-4 ^	1.52 [1.52]	1.35 [0.83]	1.31 [0.78]	0.3450
FNI	0.33 [0.42] **,***	0.19 [0.23] **	0.15 [0.22] ***	0.0006
APRI ^	0.31 [0.37] ***	0.26 [0.19] *	0.21 [0.14] ***,*	0.0023

**Table 2:** Bivariate correlations between serum ferritin concentrations and clinical laboratory parameters, inflammatory and liver indexes (WHR = waist-to-hip ratio; AMMi = indexed appendicular muscle mass; HOMA-IR = Homeostatic Model Assessment for Insulin Resistance; ASAT = aspartate aminotransferase; ALAT = alanine aminotransferase; GGT = gamma glutamyl transpeptidase; SHBG = sex hormone-binding globulin; MCV = mean corpuscular volume; MCH = mean corpuscular hemoglobin; MCHC = mean corpuscular hemoglobin concentration; SIII = systemic immune-inflammation index; FLI = Fatty Liver Index; ION = Index of NASH (non-alcoholic steatohepatitis); FNI = Fibrotic NASH Index; APRI = ASAT-to- Platelet Ratio Index; data are correlation coefficient r [95% confidence interval (CI)]).

Parameter	r [95% CI]	*p*
Clinical and anthropometric parameters		
Age (years)	0.18 [−0.30; −0.06]	0.0028
Diabetes duration (years)	−0.29 [−0.40; −0.17]	<0.0001
Diastolic BP (mmHg)	0.18 [0.06; 0.30]	0.0029
Sex (F = 1/M = 2)	0.41 [0.30; 0.50]	<0.0001
Waist circumference (cm)	0.16 [0.04; 0.28]	0.0077
WHR	0.18 [0.06; 0.29]	0.0034
AMMi	0.34 [0.23; 0.44]	<0.0001
Laboratory parameters		
C-peptide (ng/mL)	0.28 [0.16; 0.39]	<0.0001
HOMA-IR	0.28 [0.16; 0.39]	<0.0001
Albumin (g/dL)	0.19 [0.07; 0.30]	0.0021
ASAT (U/L)	0.26 [0.15; 0.37]	<0.0001
ALAT (U/L)	0.34 [0.23; 0.44]	<0.0001
GGT (U/L)	0.40 [0.29; 0.49]	<0.0001
Direct bilirubin (mg/dL)	0.27 [0.15; 0.38]	<0.0001
SHBG (nmol/L)	−0.17 [−0.28; −0.05]	0.0059
Creatinine (mg/dL)	0.22 [0.10; 0.34]	0.0002
Red blood cell count (10^6^/μL)	0.23 [0.11; 0.35]	0.0001
Hemoglobin (g/dL)	0.40 [0.30; 0.50]	<0.0001
Hematocrit (%)	0.36 [0.24; 0.46]	<0.0001
MCV (fL)	0.22 [0.10; 0.34]	0.0002
MCH (pg)	0.31 [0.20; 0.42]	<0.0001
MCHC (g/dL)	0.34 [0.23; 0.44]	<0.0001
Platelet count (10^3^/μL)	−0.18 [−0.30; −0.06]	0.0028
SIII	−0.13 [−0.25; −0.01]	0.0293
Liver steatosis and fibrosis markers		
FLI	0.19 [0.07; 0.31]	0.0015
ION	0.31 [0.20; 0.42]	<0.0001
FNI	0.26 [0.14; 0.37]	<0.0001
APRI	0.23 [0.11; 0.34]	0.0001
Liver Risk Score	0.32 [0.20; 0.42]	<0.0001

**Table 3:** Multivariable correlations between (**a**) ferritin and clinical and laboratory parameters, (**b**) Liver Risk Score, (**c**) fibrosis index FNI, and (**d**) steatosis index ION with clinical and laboratory parameters (ASAT = aspartate aminotransferase; AMMi = indexed appendicular muscle mass; BMI = body mass index; HbA1c = glycated hemoglobin; HOMA-IR = Homeostatic Model Assessment for Insulin Resistance; GGT = gamma glutamyl transpeptidase; eGFR = estimated glomerular filtration rate; SE = standard error; CI = confidence interval).

Parameter	Standardized β coefficient (SE)	95% CI	t Ratio	*p*
a. Serum ferritin (R^2^: 0.344, *p* < 0.001)				
Diabetes duration	−0.140 (1.4426)	−6.441; −0.825	−2.548	0.011
ASAT	0.204 (0.915)	0.363; 3.968	2.366	0.019
GGT	0.129 (0.178)	0.036; 0.737	2.169	0.031
Hemoglobin	0.165 (5.163)	3.386; 23.722	2.625	0.009
AMMi	0.139 (48.304)	6.674; 196.925	2.107	0.036
b. Liver Risk Score (R^2^: 0.400, *p* < 0.001)				
Ferritin	0.203 (0.001)	0.001; 0.003	3.783	<0.001
Direct bilirubin	0.207 (0.757)	1.448; 4.428	3.883	<0.001
Haptoglobin	−0.145 (0.120)	−0.581; −0.109	−2.875	0.004
HbA1c	0.437 (0.089)	0.574; 0.923	8.431	<0.001
BMI	0.195 (0.014)	0.024; 0.080	3.673	0.001
AMMi	0.112 (0.461)	0.008; 1.823	1.986	0.048
c. FNI (R^2^: 0.329, *p* < 0.001)				
Ferritin	0.198 (0.000)	0.000; 0.001	3.367	<0.001
Haptoglobin	−0.131 (0.018)	−0.080; −0.010	2.507	0.013
HOMA-IR	0.122 (0.009)	0.000; 0.035	1.996	0.047
GGT	0.305 (0.000)	0.001; 0.002	5.454	<0.001
d. ION (R^2^: 0.272; *p* < 0.001)				
Age	−0.229 (0.113)	−0.628; −0.183	−3.583	<0.001
Ferritin	0.189 (0.007)	0.008; 0.036	3.126	0.002
BMI	0.328 (0.160)	0.599; 1.227	5.719	<0.001
eGFR	−0.306 (0.054)	−0.372; −0.157	−4.855	<0.001

## Data Availability

The datasets used and analyzed during the current study are available from the corresponding author upon reasonable request.

## Supplementary Materials

The following supporting information can be downloaded at: https://www.mdpi.com/article/10.3390/medicina61091518/s1, Figure S1. a. Liver Risk Score and b. FNI four patient categories groups according to the serum ferritin and haptoglobin levels (group 1: hyperferritinemia and normal haptoglobin, group 2: hyperferritinemia and high haptoglobin levels, group 3: normal/low ferritin and normal haptoglobin levels, group 4: and normal/low ferritin levels and high haptoglobin; data are mean ± SE). Table S1. Relevant concomitant therapy in study patients (DPP4 = Dipeptidyl peptidase 4; GLP-1 R= Glucagon-like peptide-1 receptor; SGLT2 = sodium-glucose transport protein 2; EPL = essential phospholipids; OTC= over the counter supplements).
