# Supplementary material for: Hyperferritinemia and the Risk of Liver Fibrosis and Liver-Related Events in Patients with Type 2 Diabetes Mellitus and Metabolic Dysfunction-Associated Steatotic Liver Disease
Source: Medicina (Kaunas). 2025 Aug 24;61(9):1518. doi: 10.3390/medicina61091518 (PMC12471581; doi:10.3390/medicina61091518)
Supplement: Supplementary file 1 [file medicina-61-01518-s001.zip › medicina-3781069-supplementary.pdf]

Figure S1: a. Liver Risk Score and b. FNI four patient categories groups according to the serum ferritin and haptoglobin levels (group 1: hyperferritinemia and normal haptoglobin, group 2: hyperferritinemia and high haptoglobin levels, group 3: normal/low ferritin and normal haptoglobin levels, group 4: and normal/low ferritin levels and high haptoglobin; data are mean±SE).

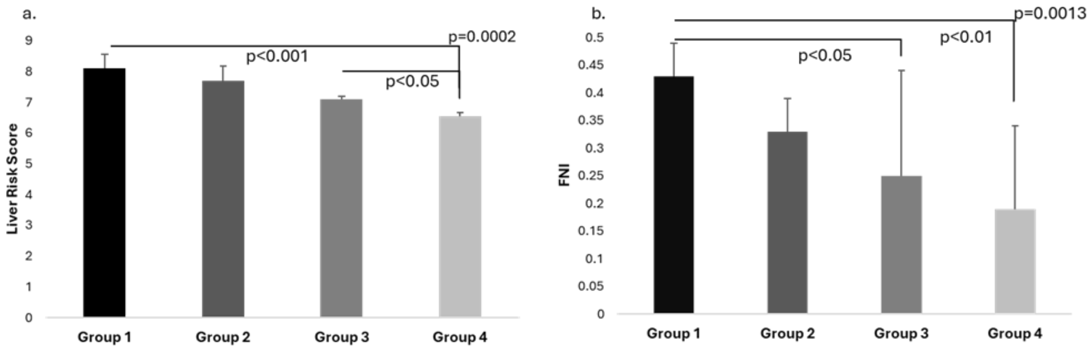

Suppl. Table S1. Relevant concomitant therapy in study patients (DPP4=Dipeptidyl peptidase 4; GLP-1 R= Glucagon-like peptide-1 receptor; SGLT2=sodium-glucose transport protein 2; EPL=essential phospholipids; OTC-over the counter supplements).

Concomitant medication	Number (%) of patients
Antihyperglycemic medication	
Metformin	266 (98.2%)
DPP4 inhibitors	20 (7.4%)
GLP-1 R agonists	94 (34.7%)
SGLT2 inhibitors	65 (24.0%)
Sulfonylureas	30 (11.1%)
Insulin	66 (24.4%)
Diet	2 (0.7%)
Liver-related therapy	
Ursodeoxycholic acid	3 (1.1%)
OTC containing EPL	38 (14.0%)
OTC containing silymarin	75 (27.7%)
OTC containing EPL+silymarin	19 (7.01%)
Other plant-based OTC	4 (1.5%)
Iron-related therapy	
Ferrous sulfate	2 (0.7%)
OCT multivitamin/multimineral complex containing small amounts of iron	3 (1.1%)
